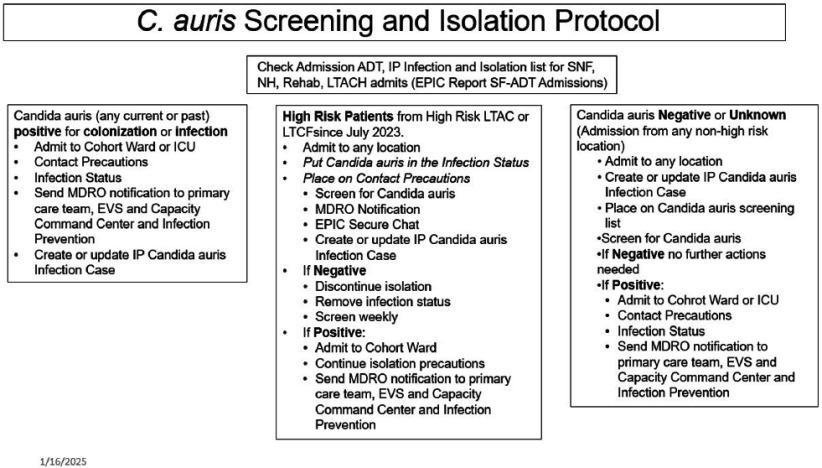# Candida auris Screening and Isolation in a Tertiary Medical Center, 2019-2024

**DOI:** 10.1017/ash.2025.426

**Published:** 2025-09-24

**Authors:** Risa Webb, Sheila Fletcher, Melinda Grubb

**Affiliations:** 1 University of Mississippi Medical Center; 2 University of Mississippi Medical Center; 3 The University of Mississippi Medical Center

## Abstract

**Background:** Candida auris(CA) first recognized in the US in 2013, can be resistant to all major antifungal agents limiting treatment options. To decrease its spread, guidelines indicate patients with CA, if admitted to hospital, should be placed in isolation and considered positive indefinitely. Screening around newly identified patients is recommended. **Methods:** We review CA history in our facility, including colonizations, infections and screening/isolation protocols from 2019-2024. **Results:** In late 2019,a patient with a CA infection was transferred to our hospital. It was late 2022, before two additional patients with CA were admitted to our faciility from a long-term acute care facility (LTAC) that had a newly recognized CA cluster of cases/colonizations. Screening in our facility did not identify additional cases/colonizations (n =49) at that time. Patients with known infection or colonization were placed in contact isolation. Additional LTAC/LTCFs were recognized from which patients with CA were routinely identified and admitted to our facility Patients from these facilities were deemed high risk (HRP) and were preemptively placed in isolation and screened for CA. From March, 2023 through August 2024,patients with CA or HRPs were placed on a cohorted ward in contact isolation. Cohorted isolation was continued on high risk but screening negative patients until three screening tests were negative and they were no longer at the high risk LTAC/LTCF. Providers were notified by email or through electronic record of patients status and reminded of infection control measures to follow. Any patient with CA had their electronic record flagged for contact isolation in the event of readmission. Screening specimens for colonization were sent to an outside laboratory until August, 2024 when in-house testing became available. With in-house testing, screening results became available in 1-2 days rather than 3-4 days. A new protocol was started and only placed patients with known positive cultures in the cohorted ward. (see image) HRPs are placed in isolation wherever in the hospital they are admitted until screening results are known. If results are positive for CA they are transferred to the cohorted ward. Additionally rooms are cleaned with appropriate disinfectants for surfaces and floors. From December, 2022, through August, 2024 we identified 22 unique patients with cultures from clinical isolates. Specimens included nine cultures from blood, including three of hospital onset. Three cultures were from wounds, one was hospital onset. Other cultures were 1 from bone, 1 from pleural fluid and 9 from urine. 106 patients were identified as colonized with screening. **Conclusion:** Screening, isolation and cohorting have all been tools for managing CA in our facility. Only three hospital onset CA bacteremias have been identified with those protocols.

**Figure:**